# Supplementary material for: Bayesian refinement of protein structures and ensembles against SAXS data using molecular dynamics
Source: PLoS Comput Biol. 2017 Oct 18;13(10):e1005800. doi: 10.1371/journal.pcbi.1005800 (PMC5662244; doi:10.1371/journal.pcbi.1005800)
Supplement: S1 Archive — (BZ2) [file pcbi.1005800.s011.tar.bz2 › gromacs_4.62_bayesian_saxs_refi/share/html/online/do_dssp.html]

do\_dssp


| do\_dssp Main Table of Contents |

**VERSION 4.6  
Sat 19 Jan 2013**

---

### Description

do\_dssp
reads a trajectory file and computes the secondary structure for
each time frame
calling the dssp program. If you do not have the dssp program,
get it from http://swift.cmbi.ru.nl/gv/dssp. do\_dssp assumes
that the dssp executable is located in
/usr/local/bin/dssp. If this is not the case, then you should
set an environment variable DSSP pointing to the dssp
executable, e.g.:

setenv DSSP /opt/dssp/bin/dssp

Since version 2.0.0, dssp is invoked with a syntax that differs
from earlier versions. If you have an older version of dssp,
use the -ver option to direct do\_dssp to use the older syntax.
By default, do\_dssp uses the syntax introduced with version 2.0.0.
Even newer versions (which at the time of writing are not yet released)
are assumed to have the same syntax as 2.0.0.

The structure assignment for each residue and time is written to an
.xpm matrix file. This file can be visualized with for instance
xv and can be converted to postscript with xpm2ps.
Individual chains are separated by light grey lines in the .xpm and
postscript files.
The number of residues with each secondary structure type and the
total secondary structure (-sss) count as a function of
time are also written to file (-sc).

Solvent accessible surface (SAS) per residue can be calculated, both in
absolute values (A^2) and in fractions of the maximal accessible
surface of a residue. The maximal accessible surface is defined as
the accessible surface of a residue in a chain of glycines.
**Note** that the program g\_sas can also compute SAS
and that is more efficient.

Finally, this program can dump the secondary structure in a special file
ssdump.dat for usage in the program g\_chi. Together
these two programs can be used to analyze dihedral properties as a
function of secondary structure type.

### Files

| option | filename | type | description |
| --- | --- | --- | --- |
| **-f** | traj.xtc | Input | Trajectory: xtc trr trj gro g96 pdb cpt |
| **-s** | topol.tpr | Input | Structure+mass(db): tpr tpb tpa gro g96 pdb |
| **-n** | index.ndx | Input, Opt. | Index file |
| **-ssdump** | ssdump.dat | Output, Opt. | Generic data file |
| **-map** | ss.map | Input, Lib. | File that maps matrix data to colors |
| **-o** | ss.xpm | Output | X PixMap compatible matrix file |
| **-sc** | scount.xvg | Output | xvgr/xmgr file |
| **-a** | area.xpm | Output, Opt. | X PixMap compatible matrix file |
| **-ta** | totarea.xvg | Output, Opt. | xvgr/xmgr file |
| **-aa** | averarea.xvg | Output, Opt. | xvgr/xmgr file |

### Other options

| option | type | default | description |
| --- | --- | --- | --- |
| **-[no]h** | bool | no | Print help info and quit || **-[no]version** | bool | no | Print version info and quit || **-nice** | int | 19 | Set the nicelevel || **-b** | time | 0 | First frame (ps) to read from trajectory || **-e** | time | 0 | Last frame (ps) to read from trajectory || **-dt** | time | 0 | Only use frame when t MOD dt = first time (ps) || **-tu** | enum | ps | Time unit: fs, ps, ns, us, ms or s || **-[no]w** | bool | no | View output .xvg, .xpm, .eps and .pdb files || **-xvg** | enum | xmgrace | xvg plot formatting: xmgrace, xmgr or none || **-sss** | string | HEBT | Secondary structures for structure count || **-ver** | int | 2 | DSSP major version. Syntax changed with version 2 |

---

http://www.gromacs.org  
gromacs@gromacs.org
